# Pesticides vs. Biopesticides: From Pest Management to Toxicity and Impacts on the Environment and Human Health

**DOI:** 10.3390/toxics11120983

**Published:** 2023-12-04

**Authors:** Gabriel Mihăiță Daraban, Raluca-Maria Hlihor, Daniela Suteu

**Affiliations:** 1 “Cristofor Simionescu” Faculty of Chemical Engineering and Environmental Protection, “Gheorghe Asachi” Technical University of Iasi, 73 Prof.dr.docent D. Mangeron Blvd., 700050 Iasi, Romania; darabangabrielmihaita@yahoo.com; 2 Faculty of Horticulture, “Ion Ionescu de la Brad” Iasi University of Life Sciences, 3 Mihail Sadoveanu Street, 700490 Iasi, Romania

**Keywords:** pesticides, biopesticides, environmental and human health impacts, plant extracts, toxicity

## Abstract

The environmental pollution that occurs in direct response to the widespread use of man-made/conventional pesticides results from many chemicals that require a long period of time, often decades, to degrade. The synthetic nature of pesticides also harms animals, beneficial insects, microorganisms, and plants, as well as humans. Fortunately, however, there are many natural pesticides, the so-called biopesticides, that are also effective against pests and more importantly, do not interfere with the well-being of ecosystems. Consequently, most biopesticides are safer for use around people and pets than man-made pesticides because, for example, they can be easily washed away from fruits and vegetables. The natural habitat is a rich resource with a wide selection of plants, many of which are also used to treat diseases in humans, animals, and plants. Out of concern for public health, environmental safety, and the stringent regulation of pesticide residues in agricultural commodities, the use of biopesticides is becoming increasingly important, but questions regarding potential pest resistance to these products may arise, just as is the case with conventional pesticides. Therefore, the performance and potential role of biopesticides in the management of plant pests should be prioritized due to their sustainability and importance to human and environmental welfare. In this review, we propose to highlight a scenario in which we discuss in detail the main constraints posed by the use of pesticides compared to biopesticides, starting with issues regarding their definition and continuing on to issues related to their toxicity and their impact on the environment and human health.

## 1. Introduction

The significant contribution of pesticides in regards to the marked improvement in the productivity of modern agriculture, along with their successful implementation in agricultural applications, is widely recognized as being of utmost value in regards to plant protection in developing countries, as the development of industrialized farming practices would otherwise have been unattainable [1]. Across the EU today, more than 333,000 tons of pesticides are marketed annually, and more than 450 active substances are currently approved for use (as of 2021) [2]. Overall, in 2020, the United States was the world’s largest consumer of pesticides, utilizing approximately 407.8 thousand metric tons, followed by Brazil, with a consumption of 377.2 thousand metric tons. Globally, pesticide intake totaled 2.66 million metric tons in 2020. Between 1990 and 2010, the global consumption of pesticides applied in farming practices expanded by more than 50%. However, since then, consumption has remained relatively stable, falling slightly from 2.68 million metric tons in 2011 to 2.66 million metric tons in 2020 [3].

The elimination of pesticides use is associated with a major decline in fruit, vegetable, and cereal production. Therefore, pesticides are widely acknowledged to play a crucial role in mitigating crop diseases and boosting crop yields worldwide [4].

In 1962, Rachel Carson released the book *Silent Spring*, alerting the public to the issues that the irresponsible handling of pesticides can create. This book triggered widespread environmental and human health concerns regarding the impact of pesticides [5]. Most national and intergovernmental authorities have strongly agreed that Integrated Pest Management (IPM) will be the forthcoming officially approved framework for assessing crop protection. Thus, since 2014, an EU Directive has obliged all agricultural producers in the EU to implement the general rules of IPM. Most guidance documents define IPM as a systematic and holistic “approach” or “strategy” to control plant pests using available methods, with a minimum reliance on synthetic pesticides. The intent of employing IPM is not to eliminate pests, but to effectively manage them while keeping their populations below economically damaging levels. Implementation of this strategy would not only reduce the toxic exposure of farmers, consumers, and the environment, but also minimize the damage from pesticide-resistant pests [6].

In contrast, there is also another class of pesticides, known as biopesticides. This class involves natural substances or substances produced by nature, especially by living organisms such as plants, fungi, bacteria, etc. [7,8,9,10,11]. Arable farming, crop maintenance, and storage of grain products, and thus the protection of human health and the environment, have been daily tasks throughout history. The resources available for this purpose were scarce, but natural, and formed the framework for the application of spontaneous herbs to preserve health and control crop and storage pests. It is assumed that approximately 250,000 to 500,000 different plant species grow on planet Earth [12], of which 1–10% are used by humans and other living things for food and medicine [13]. The International Union for Conservation of Nature (IUCN) and the World Wide Fund for Nature (WWF) report that between 50,000 and 80,000 species of flowering plants are known to be used for medicinal purposes worldwide. Of these, an estimated 15,000 species are at risk of extinction due to overexploitation and habitat depletion, and 20% of wild species have already been nearly exhausted due to human population growth and plant intake [14].

Still, the success of pesticide efficacy is at risk due to the evolution of pathogens, weeds, and resistant insect pests. Conventional pesticides target specificity to reduce non-target impacts on the environment, but this specificity also means that resistance is more developmentally accessible to target pests. Pesticide resistance consequently constitutes an intriguing model case of rapid evolution in the face of strong selective pressures [15]. The current context for the use of biopesticides is largely represented by *Bacillus thuringiensis* (Bt), but as resistance to Bt-based products begins to emerge, additional measures to combat biopesticide resistance are needed [16].

In the literature, species of spontaneous flora exhibiting pest control capabilities are often neglected, although in recent decades, there has been an increasing amount of research presenting the “bio” alternatives to partially or completely substitute synthetic pesticides with biopesticides. On the other hand, Cappa et al. [17] reported a percentage increase in the total number of scientific publications mentioning the name “pesticide” relative to those mentioning “biopesticide” in the Web of Science (WoS) database over the period of 1985–2021. Moreover, the total number of scientific publications referring to the term “biopesticide” is quite low compared to those referring to the term “pesticide” (4262 vs. 133,462 on WoS) during this period. Giraldo-Rivera et al. [18] showed that the proportion of articles reporting on “botanical insecticides/pesticides” increased from 0.94% in 1989 to 8.81% in 2000, reaching 40.12% in 2010, showing a fivefold increase. The period of 2010–2018 recorded a considerable expansion of articles on the topic, accounting for 59.81% of the total publications found in the Scopus database over the period of 1990–2018.

**Figure 1:**
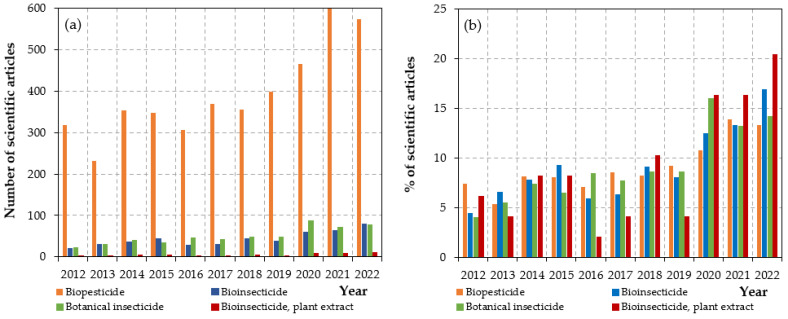
Graph showing the number of published literature articles (**a**) and their percentage in relation to the total number of articles (**b**) reporting data on “biopesticide” application versus those reporting data on “bioinsecticide/botanical insecticide/bioinsecticide; plant extract” application over the period of 2012–2022.

Figure 1 proposes an assessment of the number of articles (a) vs. the percentage of articles in relation to the total number of articles (b) in the literature, identifying scientific publications reporting the term “biopesticide” in relation to those highlighting the terms “bioinsecticide”, “botanical insecticide”, and “bioinsecticide; plant extract” during the period of 2012–2022. The PubMed^®^ database was selected to retrieve the literature publications covering our objectives. Two of the authors (G.M.D., R.M.H.) conducted an independent assessment of the scientific articles identified as reporting the above terms. Articles that were considered appropriate were included on the basis of title and abstract, and if they did not meet all eligibility criteria, the full text was examined for further evaluation.

Considering the database under assessment for the period of 2012–2022, a total of 24,676 scientific articles were analyzed for the term “biopesticide”, of which 4321 were identified as relevant; a total of 522 scientific articles were analyzed for the term “bioinsecticide”, of which 474 were identified as relevant; a total of 793 scientific articles were reviewed for the term “botanical insecticide”, of which 545 were identified as relevant; and a total of 54 scientific articles were reviewed for the term “bioinsecticide; plant extract”, of which 49 were identified as relevant (Figure 1a). On the other hand, if we consider the percentage of scientific articles published during this period, calculated in relation to the total number of articles related to a specific term, we observe that in 2022, the highest percentage—20.41%—is identified for the terms “bioinsecticide; plant extract”, with an increase of 14.29% compared to the number noted in 2012, even if the number is very low (Figure 1b).

The heightened scientific attention to the complex field of biopesticides/bioinsecticides indicates a considerable expansion of interest expressed by scientists, the public, and government authorities in sustainable, environmentally friendly commodities, with biopesticides/bioinsecticides being considered as environmentally sustainable products. The above issues point to the upward trend of scientific publications in different databases and the current strong interest in this topic.

All of these factors emphasize the relevance and the importance of studies on the replacement of synthetic pesticides with green and up-to-date biopesticide/bioinsecticide products. Therefore, we propose a systematic review focusing on pillars that will serve as comparisons for sustainable pesticide replacement with biopesticides, such as: (i) the contribution and impact of synthetic pesticides compared to biopesticides (with emphasis on, but not limitations requiring, those of plant origin,); (ii) the potential, applicability, and classification of biopesticides; (iii) the challenges of introducing biopesticides in the context of sustainable Integrated Pest Management (IPM) strategies; and (iv) the use of plant extracts/botanicals as biopesticidal/bioinsecticidal products.

## 2. The Contribution of Synthetic Pesticides and Their Impact on the Environment and Human Health

### 2.1. Defining Pesticides

Pesticides are chemical substances designed to either kill or delay the growth of insects, rodents, fungi, and weeds that interfere with or cause damage to the growth of crops, bushes, trees, woods, and other vegetation intended for human benefit. However, all chemical pesticides are essentially toxic and constitute a long-term hazard to the environment and humans via their resistance in nature or in tissues. Most pesticides are non-specific and can potentially kill life forms that are harmless or beneficial [19,20,21].

The Food and Agriculture Organization of the United Nations (FAO), using a broad definition, considers that: “A pesticide is defined as any substance or mixture of substances intended for the prevention, destruction or control of any pest, including vectors of human or animal diseases, undesirable species of plants or animals which cause damage during the production, processing, storage, transport or commercialisation of food, agricultural products, wood and wood products or animal feed or any substance which may be administered to animals for the control of insects, arachnids or other pests on or in their bodies. The term also includes substances intended for use as a plant growth regulator, defoliant, desiccant or agent for thinning or preventing premature fruit drop and substances applied to crops before or after harvest to protect commodities from damage during storage and transportation” [22].

**Figure 2:**
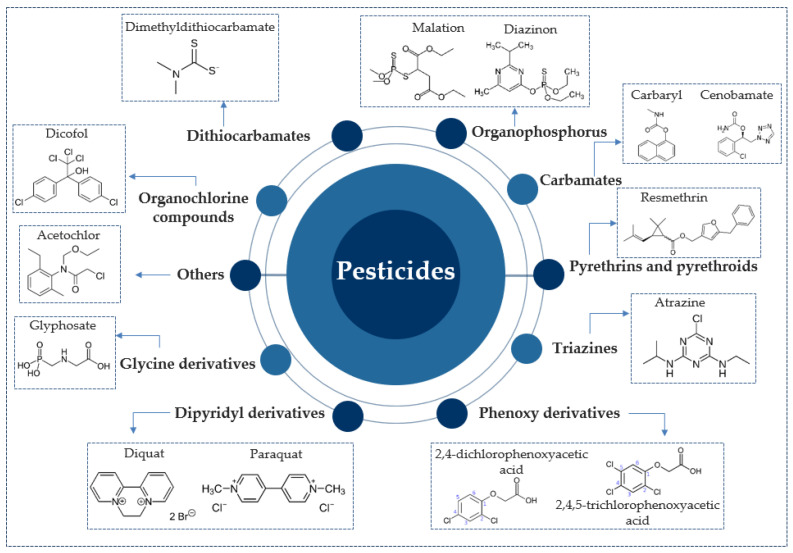
Schematic view of the main classes of pesticides and the chemical structures of their related representatives.

Pesticides are variously referred to as herbicides, insecticides, fungicides, rodenticides, molluscicides, and nematicides, according to their intended use [22]. The categorization of pesticides can also be determined by their chemical structure: organophosphorus, carbamates, organochlorides, pyrethrins and pyrethroids, benzoic acids, triazines, phenoxyacetic acid derivatives, dipyridyl derivatives, glycine derivatives, and dithiocarbamates [23] (as indicated in Figure 2).

### 2.2. Toxicity, Impacts, and Risks of Pesticides in Regards to the Environment and Human Health

The use of artificial fertilizers, pesticides, and other related products featured prominently throughout the so-called “Green Revolution” and beyond. Such issues encouraged agricultural production scenarios, and apparently, everything appeared to depend on these aids in a seemingly harmonious way [24]. A key driver of the “Green Revolution” has been finding new ways to enhance and apply pesticides considered reliable to control a wide range of plant and insect pests that damage the quantity and quality of the world’s food production [10]. Nevertheless, the downside of chemically synthesized products, such as pesticides, came to the forefront once residuals began accumulating in soil, water, and plant products. Thus, the impact of pesticides on the environment began to become tangible when these were identified as contaminants, damaging the quality of the environment as a whole [24,25].

Farmers are faced with different situations when applying pesticides in the field, as they must consider which pesticides to apply for a particular pest (or several pests), along with when and how they should be applied. Excessive pesticide use usually means increased costs and reduced profits for farmers, which in turn leads to increased risk to the environment and/or public health. Fewer pesticide treatments and fewer pounds/kilograms of active ingredients applied decrease the burden placed on non-target organisms and allow beneficial organisms and biodiversity to thrive. This is why “pounds/kilograms of active ingredient applied” is not a comprehensive metric to measure changes in pesticide use, even though it is the most common metric. This metric does not account for differences in toxicity between different pesticides, nor does it take into account differences in application methods and target pests. The Heartland Health Research Alliance (HHRA) has developed a pesticide use Minimum Dataset (MDS) composed of 18 metrics, all of which work together to answer basic questions about changes in pesticide use. In general, the rates of application have decreased, but the number of pounds/kilograms applied has remained relatively stable. For example, some pesticides are applied at high rates per acre—1 or more pounds, and even up to and over 100 pounds per acre—while other pesticides that perform approximately the same function, and sometimes work much better, may be applied at a much lower rate per acre, i.e., 0.1 pound. Thus, the question arises: Is pesticide use going up, down, or staying about the same [26]?

Pesticides, while beneficial to agriculture, have proven to be dangerous to both humans and the environment. A significant proportion of these chemicals are persistent in the environment and are prone to bioaccumulation and high toxicity. However, it is estimated that under 1% of the total amount of pesticides used to control weeds and pests reaches the target pests [27]. For example, significant amounts of pesticides are lost through spraying, off-target deposition, run-off, and photodegradation, which can have unintended consequences for species, communities, or ecosystems as a whole, as well as for human health. An additional contributing point is that low concentrations of many chemicals may not produce acute detectable effects in organisms, but may possibly induce other adverse effects, such as genetic disorders and physiological alterations, resulting in reduced long-term life span [4,27,28].

In the first stage, pesticides reach the environment during treatment preparation and application. Application can occur using different spraying techniques, depending on factors such as formulation type, target pest, and timing of application. In farming practices, it is common to apply pesticides to the crop itself or to the soil. Liquid sprays are typically applied to crops using boom sprayers, tunnel sprayers, or aerial applications. In relation to soil types, pesticides can be applied as granules, injected as a fumigant, or sprayed on the soil surface, which is eventually followed by the incorporation of pesticides into the topsoil. Additionally, seeds are often treated with pesticides before sowing occurs [4,22].

After treatment, pesticides are likely to be absorbed by target organisms, degraded, or transferred to groundwater; they may also reach surface water bodies, be volatilized into the atmosphere, or enter non-target organisms through ingestion. The behavior and fate of pesticides are directly impacted by their physical and chemical properties, the soil, the site conditions, and management practices [25]. A substantial level of active pesticide substances often remains in the soil, undergoing biological and histochemical transformations, impacting microbial and enzymatic activity in the soil. Owing to the structural diversity and the assortment of degradation pathways of synthetic pesticides, it is complicated to assess enzymatic and microbiological reactions following pesticide treatment [29].

When it comes to pesticide-related safety, there is a distinction to be drawn between the terms “toxicity” and “risk”. Toxicity relates to the inherent intoxicating capacity of a product. The toxicity of a product is assessed in toxicology laboratories, and it is expressed in quantitative terms, such as LD50 or LC50 (50% lethal dose or lethal concentration, i.e., the dose or concentration at which a product will kill 50% of a reference organism) [30]. The risk (or hazard) depends not solely on the specific toxicity of a particular substance, but on the potential for exposure when it is in use. In core terms, toxicity is the potential of a substance to produce disease or even death, while risk (hazard) is a combination of toxicity and exposure. The risk (hazard) from a particular pesticide therefore depends on the toxicity of the specific product used and the amount and form of exposure experienced [22,30,31].

The associated risks of using pesticides have outweighed their benefits. Harmful effects of pesticides on non-target species, as well as impacts and risks on plant and animal biodiversity, aquatic and terrestrial food webs, and terrestrial and food ecosystems are currently increasingly highlighted [32]. While such hazards typically vary from short-term effects (e.g., skin and eye irritation, headache, dizziness, and nausea) to chronic effects (e.g., cancer, asthma, and diabetes), the risks are difficult to assess due to the associated multiple factors (e.g., time and level of exposure; type of pesticide, in terms of toxicity and persistence; and environmental characteristics of the affected areas) [33].

In order to protect public health, scientists outside the field of pesticide manufacturing design experiments so that they maximize the reliability of the outcomes reported. In the process, they also highlight and design studies so that the chemical(s) is applied at “environmentally relevant levels”. There are concerns in regards to both health impacts and environmental risks associated with pesticide exposure through various pathways (e.g., residues in food and drinking water), especially since the mode of action of pesticides is not species-specific. However, scientists are acquiring the capabilities required to track subtle adverse effects on public health that cannot be detected through traditional testing requirements and regulatory programs, including effects on neurodevelopment, IQ, and behavior; metabolic perturbations; the microbiome; epigenetics; reproduction; and other chronic diseases and conditions. A significant gap exists in the literature, as there are few published experimental studies analyzing and evaluating the effect of different sources, various messages, and target audiences on the public health evidence uptake determinants used for prevention. In the coming decades, the extent to which pesticide exposure contributes to, rather than is largely the cause of, adverse human health and environmental effects/trends will be highlighted [33,34,35].

Siviter et al. [36] demonstrated the association between pesticide use and the decline of the bee population, along with subsequent potential losses due to lack of pollination, causing reduced crop yield. The authors also identified the correlation between pesticide application and significant negative effects on learning and memory at realistic field rates for both chronic and acute application and for both neonicotinoid and non-neonicotinoid pesticides through a meta-analysis in a comprehensive literature review [36].

Muratet and Fontaine [37] showed a negative correlation between butterfly and bumblebee abundance and the use of insecticides and herbicides, while the application of Bordeaux mixture (a fungicide approved for organic use), fungicides, and slug control products was positively correlated with butterfly and bumblebee abundance. The authors propose the hypothesis that herbicides have an indirect negative impact on insects by limiting the number of resources available to them, and that Bordeaux mixture, fungicides, and slug repellents have an indirect positive impact on these insects by favoring healthier plants, possibly providing a greater level of resources for pollinators. In addition, the authors determined that the impact of pesticides varies by landscape, with the negative effect of insecticides being greater in highly urbanized areas [37].

According to a study conducted by a Lechinovski et al. [38], which looking at the toxic potential of two conventional herbicides (atrazine and glyphosate) in regards to zebrafish (*Danio rerio*), when compared to a natural herbicide, low toxicity was demonstrated for the natural herbicide at all concentrations studied. After 72 h of exposure to different concentrations of conventional herbicides (1, 65 and 5000 µg L^−1^) and the natural herbicide (0.62, 1.25 and 2.5 µg L^−1^) in aquaria (20 L), a higher number of nuclear morphological alterations, as well as significant differences in the composition of abnormalities between treatments, were observed for conventional herbicides. The natural herbicide showed a lower mutagenic potential and was less harmful to fish; therefore, it can be considered a better option for the preservation of the environment [38].

The human body can be exposed to pesticides via the inhalation of polluted air, dust, and vapors containing pesticides; by oral exposure through the consumption of contaminated food and water, and by dermal exposure through direct contact with pesticides (e.g., dermal contact). When sprayed on plant products, especially fruits and vegetables, pesticides leach into the soil and groundwater and may subsequently enter drinking water, while pesticide vapors can drift and pollute the air [39,40,41,42].

**Figure 3:**
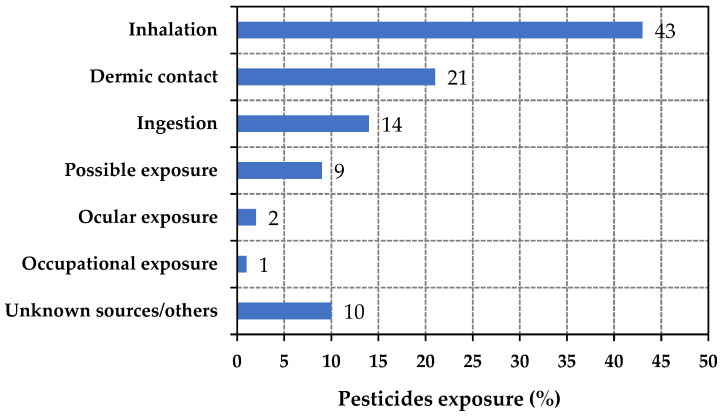
Distribution of pesticide exposure incidents in the U.S. in 2018 by type of exposure, according to data from Statista [36].

Different groups of people are affected by pesticide exposure, including workers in pesticide manufacturing plants, agricultural fields, household pest control, and greenhouses (e.g., applicators, mixer loaders, etc.). The highest likelihood of exposure to pesticides in the work area is at the time of production and formulation, as the risk is very high during this activity. In the production site, the likelihood of hazard is high because more hazardous chemicals and harmful solvents are being used [43]. In agriculture, farmers and professional applicators are frequently exposed to high levels of pesticides, resulting in the potential for adverse health effects [30,44]. It is predicted that the use of pesticides for pest control leads to an estimated 26 million poisonings globally in the human population each year, accounting for at least 3 million hospital admissions and 220,000 deaths [45]. Exposure to pesticides can cause health problems including cancer, diabetes, respiratory and neurological disorders, reproductive (sexual/genital) syndromes, and oxidative stress. All of the above conditions are linked to direct exposure, the handling of pesticides, or pesticide residues on food [46,47]. Figure 3 shows the distribution of pesticide exposure incidents reported to the U.S. National Pesticide Information Center (NPIC) in 2018, according exposure type. In 2018, a total of 2140 pesticide exposures were reported to the NPIC. Of these incidents, 43% were due to inhalation, while 21% were due to dermal/skin contact. Occupational and ocular exposure made up the lowest percentages (1–2%), while unknown sources were estimated at 10% [48].

Both acute and chronic pesticide health effects are worthy of attention and serious concern. While the acute toxicity of most pesticides is already well established, the data regarding chronic human diseases such as cancer are not as clear [45]. Evangelou et al. [49] compiled an extensive review of the evidence available in the literature regarding quantifying the risk diabetes for a wide range of pesticides. The overview included data of over 300,000 participants from more than 10 countries and 3 continents. The authors’ analysis by pesticide type showed that exposure to pesticides resulted in an increased risk of diabetes caused by exposure to DDE, heptachlor, HCB, DDT, and trans-nonchlor, or chlordane. The findings from this review, in addition to any potential biases derived from the synthesis of observational studies that might amplify the summary risk estimate, provided supporting evidence that pesticide exposure increases the risk of type 2 diabetes [49].

Multiple studies have found a direct association linking agricultural activity or pesticide exposure of farmers with respiratory symptoms (such as chronic cough, wheezing, and phlegm), asthma and chronic bronchitis, and impaired lung function. Therefore, Mamane et al. [50] suggest that occupational exposure to pesticides poses an increased risk of respiratory symptoms, asthma, and chronic bronchitis, but the causal relationship is still under debate [50].

Hlihor et al. [51] estimated consumer exposure to pesticide residues on tomatoes by calculating the long-term risk based on the hazard quotient (HQ) and the hazard index (HI), taking into account the behavior of seven fungicides and five insecticides applied to tomatoes in multiple treatments. It was determined that when pesticides were applied to tomatoes in more than one treatment, the pesticide chlorothalonil, which exceeded the MRL at harvest, exhibited an HQ value above 1 for children in both the recommended and double-dose treatments. Furthermore, the HI values also exceeded the limit of 1. These results revealed that the intake of tomatoes with multiple pesticide residues could cause non-cancer adverse effects on human health [51].

Assessing the risk posed by the impact of pesticides on either human health or the environment is therefore not an easy or particularly accurate process, due to differences in exposure times and levels, the types of pesticides used (in terms of toxicity and persistence), and the environmental characteristics of the areas in which pesticides are typically applied [21]. For example, the use of appropriate and properly stored spraying equipment and ensuring that all necessary precautions are taken at all stages of pesticide handling could reduce human exposure to pesticides, while their potentially adverse environmental effects would also be kept to a minimum [21]. Alternatively, the use of biopesticides as natural and friendly products can provide crop protection for a wide spectrum of pests and pathogens in an environmentally safe mode. Biopesticides can be a potentially beneficial alternative to the reduce the risks posed by synthetic pesticides to the environment and human health [9,52,53,54]. According to a report issued by the California Department of Pesticide Regulation (DPR) and the California Department of Food and Agriculture (CDFA), there are several biopesticides alternatives (derived from natural materials) to conventional pesticide chlorpyrifos for different types of crops and their pests, e.g., *Chromobacterium subtsugae*, Strain PRAA4-1T and spent fermentation media, Azadirachtin 4.5%, clarified hydrophobic extract of neem oil 70%, *Beauveria bassiana* Strain GHA 11.3%, Kaolin (95%), and many others [55].

## 3. Biopesticides: Alternatives to Synthetic Pesticides and Their Integration into Sustainable Agriculture

### 3.1. Defining Biopesticides and Regulatory Aspects

Numerous collaborative initiatives have been undertaken over the past three decades to address pesticide—particularly insecticide—exposure and risks to the environment and human health. The demand exists for selective and safe insecticides that preserve natural predators and non-target organisms. Some conventional pesticides have been replaced by newer, “bio-rational” pesticides, so-called biopesticides or “low or reduced risk” pesticides [19,56,57].

In the United States, the Environmental Protection Agency (EPA) has defined biopesticides (or biological pesticides) as pesticides derived from natural materials (e.g., animals, plants, bacteria, and certain minerals) [58], and at the European Union (EU) level, the European Environment Agency (EEA) has defined biopesticides as natural biological agents used to destroy pests by producing specific biological effects rather than chemical poisoning, or as a form of pesticide based on microorganisms or natural products that are naturally occurring, do not harm humans, and have minimal impact on the environment, but are classified as active substances under EU regulations (in the context of Plant Protection Products, PPPs) [59]. The FAO (Food and Agriculture Organization of the United Nations) and WHO (World Health Organization) regard biopesticides as an overarching broad concept generally applied to natural products derived from nature, such as microorganisms or plants or semiochemical substances, which can be designed and applied in a formulation similar to a conventional chemical pesticide and which are normally used for short-term pest control [60].

In Europe, biopesticides have been regulated according to established standards along the same lines as those used for chemicals. The alignment of requirements and the interpretation of registration data for biopesticides occurred with the elaboration of the Council Directive 91/414/EEC concerning the placing of plant protection products on the market and the Biocidal Products Directive 98/8/EC, covering requirements for rodenticides, insecticides, oils and extracts, naturally occurring substances, pheromones, and microorganisms, including viruses and fungi [61]. Since the adoption of Council Directive 91/414/EEC, which was subsequently replaced by Regulation (EC) No 1107/2009, any plant protection product (hereafter referred to as pesticide) must undergo a rigorous evaluation process to demonstrate that it is safe before being placed on the market in any member state of the European Union [62].

For registration purposes in the United States, the Environmental Protection Agency (EPA) divides pesticides into two broad classes: conventional chemical pesticides and biochemical and microbial pesticides. Bio-based products generally belong to the latter class, and the EPA has detailed specific testing requirements for registration, considering the “Pesticide Assessment Guidelines—Subdivision M: Biorational Pesticides” [63]. Compared to synthetic pesticides, biochemical pesticides are of natural origin and by their mode of action, are considered non-toxic. For example, insect pheromones and plant growth regulators such as auxins and gibberellins are defined as biochemical pesticides. Nevertheless, plant-extracted pesticides, although of natural origin, are not strictly non-toxic. The EPA has promoted biopesticides as part of the low-risk pesticide policy, consented to exemptions from many of the testing requirements, and has also agreed not to set tolerance levels for many biopesticides [61].

A sector of the general public believes that natural pesticides are consistently perceived to be safer and more environmentally friendly than man-made or synthetic pesticides, and while this is largely true, it is not always the case. For example, nicotine, as a natural pesticide found in tobacco leaves and as a component of cigarette smoke, is regarded as far more toxic than most modern synthetic or artificial pesticides [64].

Biopesticides, including entomopathogenic viruses, bacteria, fungi, nematodes, or secondary plant metabolites, have complex definitions acquiring increasing significance in agricultural practice as alternatives to synthetic chemical pesticides and are an important component of many pest management programs [53], leading to sustainability in agricultural practices and safety for the environment [65,66]. Biopesticides can be considered as third-generation pesticides, as they are seen as eco-friendly products that closely resemble or are identical to chemicals produced in nature. Plants and some microorganisms produce many natural chemicals used for their own self-defense against insects and pathogens. Likewise, natural pesticides are defined as those chemicals manufactured from natural ingredients. Some commonly used natural pesticides that qualify as biopesticides are: nicotine, rotenone, neem, pyrethrins, sabadilla, fluoroacetate, ryania, *Bacillus thuringiensis* (Bt), spinosad, etc. [67].

### 3.2. Biopesticide Potential, Applicability, and Classification

The advantages of using naturally occurring pesticides for plant protection include their target-specific range, delayed mode of action, reduced persistence, low residue levels (although this does not apply to all products, e.g., spinosad), and safe use relative to conventional or synthetic pesticides [68]. These features are mutually advantageous for consumers and the environment, but strict attention must be paid to the time and manner of application, as well as to the precautions that must be strictly observed. This does not mean that they are chemical-free, only that they are derived from botanical, animal, and mineral sources. They must be used with care, however, as the chemicals in biopesticides break down more quickly (such as, e.g., copper) than those from commercial products, yet they are considered less harmful than these resources [69].

Roughly 80% of pesticides applied to protect agricultural crops enter various environmental resources as a result of runoff or uncontrolled spraying, exposing animals and humans, as well as consumers of agricultural products [70]. The majority of biopesticides show efficacy against different strains of resistant species, without evidence of cross-resistance; hence, they can potentially become important in Integrated Resistance Management (IRM) strategies [67].

Biopesticides are often preferred when compared to conventional pesticides due to their specificity to target pests, efficacy, selectivity to beneficial insects, and non-persistent characteristics in the environment [56]. However, insect control through the use of Integrated Pest Management (IPM) tools provides ideal pest management alternatives [71].

As with conventional pesticides, biopesticide products must meet state regulations regarding registration, sale, transport, use, storage, and disposal. Some natural insecticides may be used in certified organic systems, if additional governmental organic standards are met [72]. As of 2020, the US EPA recorded a comprehensive list of all active ingredients in biopesticides (biochemical and microbial) for the period 1962–2020, which included 390 compounds. However, this list does not take into account the regulatory status of the active ingredients [73]. In addition, the US EPA also provides a list of so-called PIPs (Plant Incorporated Protectants) registered between 1995 and 2020, which includes 39 PIPs active ingredients, of which 5 have been cancelled for use with the cultivars listed: active products—corn, 13; cotton, 10; soybean, 6; potato, 3; other: 2; cancelled products—corn: 5 [74]. A comprehensive database, known as the BioPesticide DataBase (BPDB), has been developed at the University of Hertfordshire, UK, which includes known naturally derived pesticides [75]. The database covers a list of 748 active substances, with details on their behavior and transfer to the environment, their ecotoxicity, and their effect on human health, as well as issues related to their approval at the international level (EU and UK) under Regulation (EC) No 1107/2009 (repealing 91/414). At the EU level, biopesticides are classified as PPPs; therefore, the active substances of biopesticide must meet the approval criteria and for the most part, the general data requirements designed for conventional chemicals [76]. In the United States, many of these biopesticides also obtain certification from the Organic Materials Review Institute (OMRI) to meet the regulations set by the National Organic Program (NOP) for use in certified organic production [77].

The main categories of biopesticides include several different formulations because of their variation in terms of the solubility of the active compound and their ability to control the pest, as well as the ease of handling and transport. Plants are capable of synthesizing a full range of structural variants that exhibit an almost equally full range of insecticidal biological activities [71,78].

Inherently distinguished from conventional pesticides, biopesticides comprise three major categories [79,80]:

- Biochemical pest control agents or biochemical pesticides (e.g., botanical pesticides, essential oils, pheromones, hormones, natural plant growth regulators, enzymes, minerals, etc.);
- Microbial pest control agents or microbiological pesticides (e.g., microorganisms);
- Macrobiological pest control agents or macrobiological pesticides (e.g., beneficial insects, parasitoids and entomopathogenic nematodes).

The US EPA identifies Plant Incorporated Protectants (PIPs) as the third category of biopesticides. They are pesticidal substances produced by plants, as well as the genetic material necessary for the plant to produce the substance [81]. PIPs can result from transgenic and non-transgenic manipulations, such as direct genome editing and seed treatment [16]. On a global scale, there is a discrepancy regarding the interpretation of the term “biopesticide”, as defined by the US EPA, which is why the International Biocontrol Manufacturers Association (IBMA) and the International Organization for Biological Control promote the use of the term “biocontrol agents” (BCA) instead of biopesticide. IBMA categorizes biocontrol agents into four groups: (1) macrobicidal agents, (2) microbial agents, (3) natural products, and (4) semiochemical agents (insect behavior modifiers) [82,83].

Biopesticides therefore have a complex classification, e.g., Integrated Pest Management (IPM) strategies, including a combination of synthetic and biological crop protection products to achieve synergies of action and lower overall use. To be included in these categories, pesticides must be naturally occurring, and if the chemical is artificially synthesized, it must be structurally identical to a naturally occurring chemical. Minor differences in the ratios of stereochemical isomers (found in the natural versus synthetic compound) will not normally preclude the classification of a chemical as bio-rational, unless one isomer is found to have significantly different toxicological properties from another isomer. Thus, the application of bio-rational active agents as an alternative control strategy results in the need to seek environmentally friendly, biodegradable, and readily available affordable pest control products [68].

**Figure 4:**
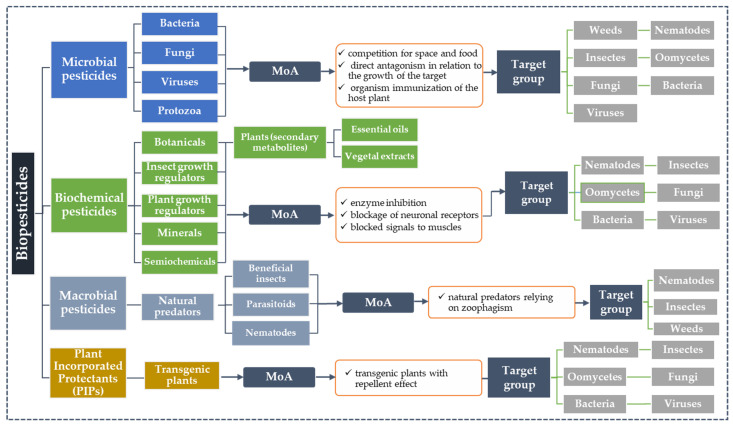
Schematic of the main categories of biopesticides, their interactions with different types of pests, and their mode of action (MoA).

There are several sources from which biopesticides can originate, each of which plays a specific or general role, depending on a number of factors and the type of pest for which it is administered. The general sources, according to kingdom, from which they come are, as previously stated: animal, plant, mineral, and microbial. Each of these main classes can in turn be sub-classified into sub-classes of compounds with pesticidal effects, depending on a number of factors. A comprehensive picture of how biopesticides might be classified under different organizations as a whole, along with their mode of action (MoA) and target group, are shown in Figure 4.

### 3.3. Challenges in Biopesticide Introduction

Sustainable pest management is a goal that can be valued via an agreement that links a series of possible approaches, namely Integrated Pest Management (IPM) [84]. Therefore, IPM objectives are associated with decreasing pest numbers on a sustainable basis and achieving cost-effective and high levels of efficacy, while at the same time ensuring that the environment continues to be safe [85]. IPM is a complete system aimed at involving multiple pest management practices, often occurring through gradual changes in practices from no IPM, to low IPM (including crop monitoring to make spraying decisions), then to medium IPM (incorporating multiple preventive practices), and finally, to high or biointensive IPM [86,87,88]. Biocontrol measures can be considered safe and profitable, but are only effective if all parts of the IPM comes together, as their efficacy is dependent largely upon a number of factors outside the farmer’s control. Therefore, farmers moving towards prevention-based and biointensive IPM solutions require a more comprehensive set of tactics, practices, and inputs that they can quickly call upon when they can no longer cope with pests [89]. If farmers adopt and perfect biointensive IPM systems, they can reduce the frequency and severity of pest population outbreaks that require control interventions [88,89]. Once that is achieved, biopesticides can become economical and reliable alternatives, but there are no expectations that they could work as well as conventional pesticides without a considerable investment in research and development, as well as industry infrastructure.

Therefore, biopesticides/biocontrol agents represent a core element of IPM, in addition to various crop protection approaches, comprised of resistance or host tolerance; good agricultural practices; reliance on natural enemies, such as predators and parasitoids; microbial pesticides; and reduced application, based on synthetic pesticides [90,91]. This approach, combined with the monitoring and early detection of pests using smart technologies such as the Internet of Things (IoT) and geographic information systems (GIS), would enable effective and sustainable crop pest management in a timely manner [92,93].

Pest control measures provide a way to keep pests below the level at which they can cause economic damage. Pest management is not about eradicating pests; it is about finding strategies that are effective and economical, ensuring that environmental damage is minimized [94,95]. IPM aims to manage crops by using several practices to keep pest levels below an economically damaging threshold. IPM has been developed as a way to control pests without relying solely on pesticides [96,97]. Integrated pest management is a systematic plan that brings together different pest management techniques into a single program. It reduces the focus on pesticides by incorporating crop control, biological, genetic, physical, regulatory, and mechanical measures [68]. Pest management relies upon the ability to properly detect pests, assess pest populations precisely, measure damage ratings, and understanding available pest management strategies or tactics that enable the pest professionals to provide insightful pest control choices. IPM has the potential to simultaneously enhance the effectiveness of pest management programs while minimizing some of the adverse impacts. Many successful IPM programs have lowered pesticide use, increasing environmental protection [68]. When an IPM program has been carried out improperly, it will result in crop damage and loss during storage due to inadequate storage structures and a lack of effective pest control strategies [98].

Although biopesticides have proven their effectiveness against a wide range of crop-specific pests, studies indicate that they are still not well accounted for in the pesticide market [84,99]. For example, the marketing of botanical pesticides takes into account the widespread availability of plants that should be easy to cultivate. In general, plants used to make biopesticide products are being cultivated for food, medicinal, or ornamental purposes, or are found naturally in forests and other uncultivated areas. The cultivation of plants for the production of biopesticides would require vast cultivated surfaces, potentially competing with food farming on agricultural land with a large area under cultivation. In some cases, farmers would choose to invest in more profitable enterprises, thereby jeopardizing food security [100]. As for the biopesticide trade, it is forecast to outgrow the chemical pesticide trade at a compound annual increase of more than 15%. The production of biopesticides is estimated to equal the production of conventional pesticides in terms of market size sometime in the late 2040s to early 2050s, but considerable variability in adoption rates, notably in regions such as Africa and Southeast Asia, contributes to explaining much of the flexibility in these projections [7]. Statista [101] made an estimate of the market value of biopesticides, predicting their value to increase from USD 1.38 billion in 2016 to USD 1.8 billion in 2021. The biopesticides market size is expected to reach about USD 2.3 billion in 2027 [101].

Despite progress and efforts to replace conventional pesticides with biopesticides in recent decades, the global share of biopesticide use is still low. The main reason why biopesticides are currently used at a low rate, despite the very promising potential results demonstrated in numerous studies, is the ability of pests to develop resistance, as with widely used synthetic pesticides. However, biopesticides remain effective when used locally for a short period of time and can complement the effects of conventional pesticides on pests. Their value lies precisely in the fact that they often act without considerable adverse effects and with high efficiency, and that they can act both alone and in synergy with conventional pesticides.

Therefore, a key constraining element in the application of botanical pesticides, for example, is the limited availability of cultivable land for the production of the required quantities of plant extracts. Also, high volumes of plant material necessary for botanical pesticide production would involve extensive capital costs related to storage and equipment. Another limiting factor in the production of botanical pesticides is the strong competition with synthetic pesticides because they are relatively simple to fabricate and to formulate, have a very long lifespan, are user-friendly, and feature proven manufacturing installations. Designing formulations of botanical pesticides is also challenging, as a plant may contain many different active compounds that vary in chemical properties [84]. This characteristic, however, could be further harnessed by a combination of multiple herbs with closely allied compounds that are synergistically efficient at combating pests [102]. Despite the low or non-existent toxicity of plants that may be used for biopesticide production, the available procedures for regulating their use in agriculture are similar to those employed for synthetic products. The registration process is expensive and includes a number of barriers, making biopesticides nearly unavailable on the market [84].

**Figure 5:**
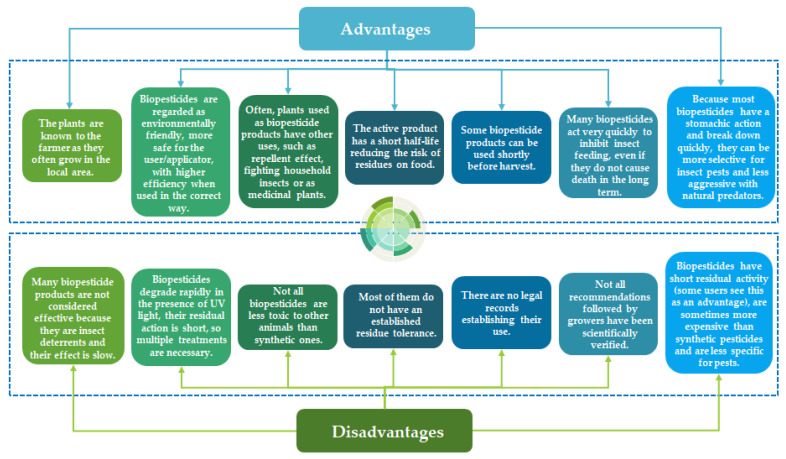
Advantages and disadvantages of biopesticides use.

Awareness among micro farmers of the usefulness of biopesticides in crop pest management is limited. Weather conditions influence the sprayability of botanical pesticides, which are highly biodegradable, particularly if used in their natural form [103]. Being readily biodegradable, the lifespan of botanical pesticides is also quite limited. Although botanical pesticides are known to be safe, certain plants with antimicrobial properties are also linked to toxicity among non-target groups. For example, rotenone, derived from *Derris* and *Lonchocarpus*, is a compound known to be toxic to mammals, fish, and insects [84]. Also, the *Tephrosia* species, which is used as a potential bioinsecticide with effectiveness against several pests, is toxic to *Clarias gariepinus* [102]. Because of these multiple challenges, most agrochemical companies are not willing to invest in biopesticide production. For example, the quality and stability of botanical pesticides depend on the type of plants used to prepare the plant extracts, the solvent system, the temperature range, and the storage conditions; in addition, the extraction of biopesticides requires the use of organic solvents whose disposal raises environmental pollution questions, as it calls for enhanced extraction and residue disposal methods [84]. Figure 5 lists some of the most significant advantages and disadvantages of implementing biopesticides for sustainable agriculture.

The philosophy and approach of the bio-based pesticide regulations require registrants to obtain approval from the expert committee prior to product registration. In regulating bio-rational pesticides, it is acknowledged that these types of pesticides are different from conventional pesticides, and due consideration will be given to the fact that many classes of control agents that rely on biopesticides may pose lower potential risks than do conventional pesticides. The most important underlying characteristics that distinguish biopesticides from conventional pesticides are the specificity of the target species, the generally non-toxic mode of action, and the natural presence of bio-radicals. These factors provide the basis for expecting many classes of biocontrol pest control agents to present a lower potential risk than conventional pesticides, supporting the testing approach required for product registration. Thus, the environmental protection laws in various states have framed a number of rules and standards to regulate the application of chemical control agents in nature [104].

From a life cycle perspective, assessing the environmental impacts and costs of biopesticides generated by the cultivation and production of the raw materials needed for biopesticide production can be very difficult to assess, as the sources used for biopesticide treatments are many and varied. For example, in the case of microorganisms, production impacts and costs are relatively low compared to other methods, both eco-friendly and conventional, but these microorganisms cannot control the full spectrum of pests, so other sources of treatments are needed. When it comes to plant-based biopesticides, there are many variables that can generate both impacts (positive or negative) and low or high costs, starting from the plant species used, the method of planting and/or harvesting, the type of processing and storage, the extraction of active ingredients, the mode of administration, the frequency and amount administered, the pest targeted, etc. Given the multitude of factors that contribute to the assessment of impacts and costs of obtaining a biopesticide, it can be concluded that they can be reduced or increased at the same time, depending on the factors listed above. In conclusion, the cost–benefit principle will be in balance with the principle of supply and demand; taking into account the impact on the environment and human health, this formula can adjust the ratio of pesticides and biopesticides used globally.

### 3.4. A New Generation of Biopesticidal Compounds: Plant Extracts/Botanicals

The extraction and isolation of target compounds form the main step in the production of plant raw materials. The final extract quality and the effectiveness of a specific method are determined by several variables, i.e., type of plant material and pre-extraction sample preparation, type of solvent, extraction technique, physicochemical conditions, etc. [105]. Extraction provides a basic route for the separation and recovery of bioactive compounds present in plants. It transforms the actual matrix into an appropriate reference sample for the subsequent analytical procedure [106]. Currently, in order to increase the extraction efficiency and selectivity of bioactive compounds, conventional extraction methods such as maceration, percolation, Soxhlet extraction, digestion, and preparation of decoctions and infusions are being replaced by modern extraction methods to meet the increasing market demand. Modern techniques use different extraction methods such as microwaves, ultrasounds, supercritical fluids, enzymes, pressurized liquids, electric fields, etc. Various studies have pointed out the possibility of combining extraction techniques (conventional and modern) as a promising technique for quickly and efficiently obtaining the desired extracts [106,107].

Botanicals can be a comprehensive source of active ingredients for the development of effective insecticides and acaricides to control agricultural crop pests. Essential oils and (crude) plant extracts are considered to be insecticides with a broad range of action, depending on the physiological characteristics of the insect species, as well as the type of plant: they can act as repellents, attractants or inhibitors of feeding or respiration, prevent host plant identification, inhibit oviposition, and reduce adult emergence through ovicidal and larvicidal effects. Among other types of bioinsecticides, botanicals are the most commonly used [108,109].

Of the plant extracts currently suggested for pest control, essential oils have been identified as prospective active ingredients for insecticidal formulations owing to their worldwide availability and relatively inexpensive nature, in addition to their alleged human health properties and environmental safety [110].

The efficacy of essential oils and plant extracts is dependent on molecules that plants synthesize as part of their built-in mechanism of protection against microbial pathogens and pests. To obtain essential oils, steam distillation is frequently applied, although they can also be derived from plants by means of fermentation, solvent extraction, and enfleurage. However, plant extracts are typically produced from dried plant material, primarily through a solid–liquid (S/L) extraction technique employing aqueous or organic solvents or mixtures thereof, e.g., acetone, ethanol, hexane, or methanol [109].

Unfortunately, few of these products have been rigorously registered due to a lack of resources and weak regulatory systems. The flora of many developing countries can be a rich source of potent and valuable plant extracts and essential oils, but the results required for a risk analysis are variable or elusive and are often not supported by scientific experiments. Thus, the potential of many spontaneous plant species remains untapped [67].

For example, Daraban et al. [111] conducted a study to test 14 plants from Romania’s spontaneous native flora for potential insect repellent and insecticidal effects on the Colorado beetle (*Leptinotarsa decemlineata* Say). Among all the studied botanicals, only three of them resulted in increased mortality (%) for adults and larvae of *Leptinotarsa decemlineata* Say after 168 h, in the following order: *Primula veris*, *Origanum vulgare*, and *Achillea millefolium*. The maximum mortality (%) was observed after treatments with crude alcoholic extracts of *Primula veris* extracted using the heat reflux extraction method in a Soxhlet apparatus, with 90% for larvae and 80% for adults, respectively [111]. Extracts from the Romanian spontaneous flora oregano (*Origanum vulgare*), horsetail (*Achillea millefolium*), wormwood (*Artemisia absinthium*), and primrose (*Primula veris*) were prepared in a preliminary study using two extraction methods (maceration and reflux extraction) in an attempt to identify their bioinsecticidal properties. Tests showed that the reflux extraction method in the Soxhlet apparatus performed best, with the solid/liquid (S/L) ratio and extraction time varying according to the type of plant: wormwood (*Artemisia absinthium*)—1 h and S/L ratio = 1/15; horsetail (*Achillea millefolium*)—2 h and S/L ratio = 1/20; oregano (*Origanum vulgare*)—2 h and S/L ratio = 1/15, and primrose (*Primula veris*)—2 h and S/L ratio = 1/10. Primrose (*Primula veris*) was found to exhibit the strongest bioinsecticidal character [112].

In developed countries, there are strict regulations regarding the use of botanical pesticides, and these are likely to be tightened further. However, this can ensure a high level of risk analysis, leading to ethical products based on essential oils and plant extracts being successfully registered [67,113].

Unlike conventional insecticides, as broad-spectrum products that affect different organisms, herbal or botanical insecticides are more selective due to the high compatibility between the active ingredients and the metabolic pathway of the targeted pest. On the other hand, they may kill pests directly, their modes of action may indirectly interfere with pest physiology and/or reproduction, or they may simply repel pests via compounds they avoid [114]. Bioinsecticides possess diverse modes of action, show efficacy against different strains of resistant species without evidence of cross-resistance, exhibit resistance to insect pests, and are considered neurotoxic poisons that act at specific target centers in the insect nervous system. Some bioinsecticides act similarly to the old neurotoxic poisons, resulting in knockdown, rapid intoxication, lack of coordination, paralysis, and death, and show a higher affinity for insect receptors than mammalian receptors. Other bioinsecticides affect specific systems, such as molting processes, metamorphosis, and the endocrine system of insects. All bio-rational or low-risk insecticides have a relatively low damaging effect on the environment and impart little or no negative consequences on non-target organisms, making them important components of integrated pest management [18,19,115,116,117].

Several classes of plant-derived chemicals (secondary metabolites) can be distinguished, including alkaloids, rotenoids, phenolic compounds, pyrethrins, oils, saponins, and others. For example, nicotine is effective against several insects (aphids, leafhoppers, thrips, and whiteflies), but its use is declining because of its high toxicity to vertebrates. Rotenone is used against mites and lice, although it has been found to be toxic to fish. Azadirachtin is also a well-known terpenoid substance that is used against stored product pests (cockroaches). Sabadilla is used against vegetable pests. Ryania is used against cockroaches, moths, thrips, and aphids. Both sabadilla and ryania are neurotoxic. Pyrethrins are used against flying insects; fortunately, their mammalian toxicity at the concentrations used is minor [118]. The nicotine, rotenone, pyrethrins, and alkaloids in sabadilla are important examples of the first generation of botanical pesticides [119]. Botanical pesticides have been shown to act specifically in the insect nervous system, to affect γ-aminobutyric acid (GABA) and sodium chloride channel closure, and to competitively combine acetylcholinesterase, the nicotinic acetylcholine receptors (nAChR), and the octopamine and tyramine receptors [120].

The literature includes a large number of studies focusing on the use of essential oils and crude plant extracts in the control of several types of pests, with favorable results. Pesticide-like chemical species found in plants have been reported to be effective against insects [121], fungi [122], bacteria [123], nematodes [124], and viruses [125]. Thus, the insecticidal effect of *Artemisia annua*, *A. absinthium*, *A. camphorata*, *A. dracunculus*, and *A. vulgaris* was investigated on the larvae of the watermelon worm, *Diaphania hyalinata* (Lepidoptera: Crambidae), a pest of *Cucurbitaceae*, as well as for their selectivity to ants of the species *Solenopsis saevissima* (Smith) (Hymenoptera: Formicidae) and the jatai bee, *Tetragonisca angustula* (Latreille) (Meliponinae). In these cases, a mortality of 42% to 96% was recorded, depending on the plant species and the type of pest being controlled [126]. Dancewicz and Gabryś [127] used *Artemisia absinthium* L. extract and essential oils, alone or mixed with other essential oils or soap solutions, in the control of peach aphids, *Myzus persicae* (Sulz.). The use of *A. absinthium* oil resulted in 80–90% mortality within 48 h of application [127]. The essential oils of three species of the genus *Artemisia* from Tunisia have been studied to reduce the contamination of stored cereals using *Tribolium castaneum*. *Artemisia absinthium* essential oil was found to have a very rapid repellent action and induced greater than 60% mortality after 24 h of application at an essential oil dose of 200 mL/L [128]. The extract and essential oils obtained from *Achillea millefolium* and *Origanum vulgare* have been studied for their repellent action against *Acrobasis advenella* (Zinck.) (Lepidoptera, Pyralidae), which is the most dangerous pest of aronia (*Aronia melanocarpa* [Michx.] Elliot) [129]. *Achillea millefolium* essential oil has been shown to exhibit contact and fumigant toxicity against adult *Tetranychus urticae* Koch. [130], while the essential oil of *Origanum vulgare* L. has been studied for its toxicity, physiology, and biochemical characteristics against the diamondback moth, *Plutella xylustella* L. (Lepidoptera: Pyralidae)—a major and cosmopolitan pest of crucifer crops [131]. Essential oils of *Origanum vulgare* L. have been studied for toxicity and physiological effects on the pyralid *Glyphodes pyloalis* Walker (Lepidoptera: Pyralidae) under controlled conditions [132]. The essential oil of another species of oregano, *Origanum glandulosum* (Desf.), has been investigated for its insecticidal activity against *Rhizopertha dominica* [133]. These results are consistent with the results obtained by Daraban et al. [134]. The authors highlighted the use of plant extracts from the spontaneous flora of Moldova (Romania) as bioinsecticides to control field pests of Leptinotarsa decemlineata. Plant extracts from oregano (*Origanum vulgare*), horsetail (*Achillea millefolium*), wormwood (*Artemisia absinthium*), and primrose (*Primula veris*) were evaluated for their antioxidant activity (for polyphenol and flavonoid content and for 2,2-diphenyl-1-picrylhydrazyl (DPPH) radical scavenging activity) and characterized by FTIR spectroscopy. To assess the bioinsecticidal properties of plant extracts, mortality (%) and neuroleptic manifestations occurring at mid monitoring period for larvae and adults of the species *Leptinotarsa decemlineata* Say were identified. The highest mortality (%) was observed after 24 h of treatment with 100% *Origanum vulgare* extracts, while the maximum effect was recorded after 48 h for larvae and after 144 h for adults [134]. The same plant extracts, *Origanum vulgare*, *Achillea millefolium*, *Artemisia absinthium*, and *Primula veris*, were also tested for bioinsecticidal activity against the bean beetle, *Acanthoscelides obsoletus*. The obtained extracts were shown to be effective in the following order: *Origanum vulgare* > *Artemisia absinthium* > *Achillea millefolium* > *Primula veris* (the last two having similar bioinsecticidal effects). The studies also showed that *Origanum vulgare* extract was much more effective than *Artemisia absinthium* in controlling the species studied, given the mortality recorded among the insects (70%) after 168 h of contact time [115].

Sales et al. [103] investigated herbal tinctures against *Fusarium guttiforme* and *Chalara paradoxa* species causing pineapple fusariosis by analyzing their antifungal activity. The authors proved that the extracts used were able to effectively inhibit the growth of *Fusarium guttiforme* by up to 46% and that of *Charala paradoxa* by up to 29%, with the extracts from *Aloe vera*, *Allium sativum*, and *Glycyrrhiza glabra* being as efficient as the synthetically produced fungicide tebuconazole (Folicur® 20EC, Hyderabad, India) [103]. The inhibition of mycelial growth of the tomato mold pathogen *Fusarium oxysporum* was achieved by using extracts from *Azadirachta indica* and *Oscimum sanctum* species, with levels reaching 100% [84]. Mycelial growth of *Penicillium digitatum*, causing gray mold on oranges, was reduced with extracts of *Azadirachta indica*, *Cerbera odollam*, and *Capsicum frutescens* by up to 90%, while extracts of *Zingiber officinale* and *Cymbopogon nardus* inhibited its growth by up to 70% in vitro. Therefore, a significant reduction in orange fruit deterioration and quality was identified by using treatments based on extracts from *A. indica*, *C. odollam*, and *C. frutescens* [135].

Crude extracts from the plant species *Allium sativum*, *Zingiber officinale*, *Curcuma longa*, and *Citrus limon*, as well as microbial antagonists of *Trichoderma* and *Paecilomyces* species, contributed to a high population decline of whiteflies and thrips, but also to a reduction of rust (*Uromyces appendiculatus*), angular leaf spot (*Phaeoisariopsis griseola*), and anthracnose (*Colletotrichum lindemuthianum*) in green beans (*Phaseolus vulgaris*) [91]. Extracts belonging to the species *Allium cepa*, *Allium sativum*, *Phyllanthus emblica*, *Curcuma zedoaria*, *Calotropis procera*, *Azadirachta indica*, and *Ocimum canum*, along with cow dung and mineral salts from cow urine, have proven their efficacy in combating tomato pests such as *Helicoverpa armigera* by reducing crop damage and enhancing productivity [136].

The examples identified demonstrate the important contributions of botanical pesticides to the management of crop pests in long-term IPM programs. Thus, it has been demonstrated that, through their activity, botanical pesticides can act against a wide range of pests; have a diverse mode of action; show potency in a variety of agroclimatic zones, seasons, and crops; and make a key contribution in increasing crop performance while preserving the environment, health, and biodiversity. Several of these bioinsecticidal products were already widely used, and their activity and mode of action are described below. Table 1 also highlights a number of physicochemical, ecotoxicological, and mode of action properties, as well as the main producers of some common botanical biopesticides or active substances with important bioinsecticidal properties, but which may or may not yet be commercialized.

## 4. Conclusions

Biopesticides are continuing to expand at a rate that exceeds that of synthetic pesticides and, when incorporated into Integrated Pest Management programs, can offer benefits such as residue management and pest resistance, along with low risks to beneficial insects or other non-target groups. Developing countries possess a huge potential for biopesticide use because production can be cheaper, labor is cheap compared to that in developed countries, and wild flora is often very abundant.

Keeping in mind the large amount of plant stock required for biopesticide manufacturing, cultivation of plants used in their production on a large scale may be conducted on marginal land which is unsuitable for agricultural crops to prevent any potential risk of food crop competition. Consideration should be given to low-cost solvent processing and the extraction of biopesticides to minimize production costs and problems regarding the landfill of waste. Small farmers should then be able to easily afford the biopesticide products in order to further introduce reliable anti-pest control agents.

Bearing in mind that biopesticides are readily biodegradable, further studies are still necessary to design products with a longer shelf life, while preserving the intended effectiveness. Research regarding the durability of botanical pesticides, particularly under field conditions, should be further pursued. To date, several products with the potential to be further developed into biopesticides have been identified, isolated, and characterized. Further research is required to ensure that the efforts made so far to identify and isolate these products have not been in vain. Barriers to the efficacy of certain products have been identified and need to be addressed, even while research continues on new products. Stakeholder collaboration remains crucial; therefore, biopesticide researchers need to work closely with industry leaders, farmers, policy makers, government officials, and other relevant stakeholders.

## Figures and Tables

**Table 1:** Physicochemical properties of some selected botanical active substances, including the target group and formulation.

Plant and/or Active Substance	Species ^a^	Type ^b^	Molecular Formula ^a^	Molecular Mass (g/mol) ^a^	Toxicity ^a^	Chemical Structure ^c^	Formulation ^b^	Product (Producer) ^b^
α-pinene	*Teucrium montanum*, *Xylopia aromatica*, etc.	Fungicide	C_10_H_16_	136.23	Mammals (oral): LD50 = 3700 mg/kg	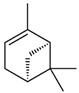	Emulsifiable concentrate to be diluted and used as a foliar spray	Timorex (BioMor, Riga, Latvija)
Capsaicin	*Capsicum* spp.	Repellent, insecticide, miticide, rodenticide	C_18_H_27_NO_3_	305.4	Mice (oral): LD50 = 47.2 mg/kg	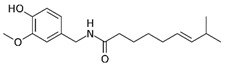	Granular, powder and liquid formulations, with capsaicin alone, or in combination with other active biopesticides	Capsaicin (Aversion Technologies Inc., Annapolis, Maryland, USA) Hot Pepper Wax Natural Insect Repellent (Hot Pepper Wax, Inc., Greenville, PA, USA) Armorex (Soil Technologies Corp., Fairfield, IA, USA)
Neem/ Azadirachtin	*Azadirachta indica*	Insecticide, fungicide, acaricide	C_35_H_44_O_16_	720.71	Rats (oral): LD50 > 5000 mg/kg	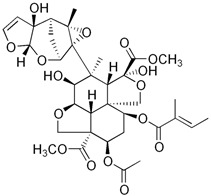	Emulsifiable concentrate applied as a spray	Azatin XL (OHP Inc., Morrisville, NC, USA) Fortune Aza (Fortune Bio Tech Pvt Ltd., Yadadri, Telangana State, India) Neemix (Certis Biologicals, Columbia, MD, USA)
Nicotine	*Nicotiana tabacum*	Insecticide	C_10_H_14_N_2_	162.23	Mice (oral): LD50 = 24 mg/kg Humans (mammary glands): 200 µmol/L/24 h	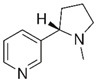	smoke-generating formulations	Nicotine 40% Shreds (Dow AgroSciences LLC, Indianapolis, IN, USA)
Piretrum	*Chrysanthemum cinerariifolium*	Insecticide, acaricide, veterinary substance	C_43_H_56_O_8_	700.9	Rats (oral): LD50 = 584–900 mg/kg	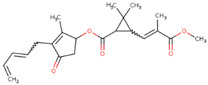	Very low volume liquids and ready-to-use sprays	Evergreen Crop Protection 60-6 (MGK, Minneapolis, MN, USA) Diatect II (Diatect International Corp., Heber City, UT, USA) Pyrethrum 5EC concentrate (Agropharm Ltd., Buckinghamshire, United Kingdom)
Ryania/ Ryanodine	*Ryania speciosa*	Insecticide	C_25_H_35_NO_9_	493.5	Humans (oral): LDLo = 143 mg/kg Mice (oral): LD50: 650 mg/kg Rats (oral): LD50 = 750 mg/kg	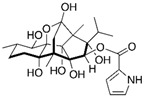	Water-dispersible powder	Natur-Gro R50 (AgriSystems International, Columbus, Ohio, USA) Ryan 50 (Dunhill Chemicals, Rosemead, CA, USA)
Rotenone	*Derris* spp. sau *Lonchocarpus* spp.	Insecticide, acaricide, veterinary substance	C_23_H_22_O_6_	394.4	Humans (oral): LDLo = 143 mg/kg Mice (oral): LD50: 2800 µg/kg Rats (oral): LD50: 60 mg/kg	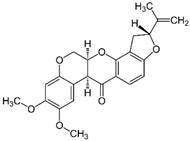	Emulsifiable concentrate or powder	Prentox Cube Powder (Prentiss Incorporated, New York, NY, USA) Vironone (Vipesco, Ho Chi Minh City, Vietnam) Derris (Nantong Shenyu Green Medicine Co., Ltd., Shanghai, China)
Sabadilla/ Veratrine (Cevadine)	*Schoenocaulon officinale* and *Veratrum oblongum*	Insecticide, miticide	C_32_H_49_NO_9_	591.7	Bees: LD50 = 12.33 UGB Mice (intra-peritoneal): LD50 = 3500 µg/kg	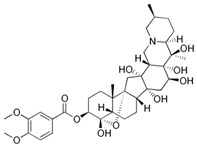	Water-soluble powders and concentrates for use as sprays	Veratran D (MGK, Minneapolis, MN, USA)

^a^ https://pubchem.ncbi.nlm.nih.gov/ (accessed on 12 February 2023), ^b^ https://sitem.herts.ac.uk/aeru/bpdb/index.htm (accessed on 15 February 2023), ^c^ https://ro.wikipedia.org/wiki (accessed on 20 March 2023).

## Data Availability

Not applicable.
